# Advances in Fluorescent Single-Chain Nanoparticles

**DOI:** 10.3390/molecules22111819

**Published:** 2017-10-26

**Authors:** Julen De-La-Cuesta, Edurne González, José A. Pomposo

**Affiliations:** 1 Centro de Física de Materiales (CSIC, UPV/EHU)—MPC, Materials Physics Center, Paseo Manuel de Lardizabal 5, E-20018 San Sebastian, Spain; julen.delacuesta@ehu.eus (J.D.-L.-C.); edurne_gonzalez001@ehu.eus (E.G.); 2 Departamento de Física de Materiales, Universidad del País Vasco (UPV/EHU), 1072 Apartado, E-20080 San Sebastian, Spain; 3 IKERBASQUE—Basque Foundation for Science, María Díaz de Haro 3, E-48013 Bilbao, Spain

**Keywords:** nanoparticles, fluorescence, optical imaging

## Abstract

Fluorophore molecules can be monitored by fluorescence spectroscopy and microscopy, which are highly useful and widely used techniques in cell biology, biochemistry, and medicine (e.g., biomarker analysis, immunoassays, cancer diagnosis). Several fluorescent micro- and nanoparticle systems based on block copolymer micelles and cross-linked polymer networks, quantum dots, π-conjugated polymers, and dendrimers have been evaluated as optical imaging systems. In this review, we highlight recent advances in the construction of fluorescent single-chain nanoparticles (SCNPs), which are valuable artificial soft nano-objects with a small tunable size (as small as 3 nm). In particular, the main methods currently available to endow SCNPs with fluorescent properties are discussed in detail, showing illustrative examples.

## 1. Introduction

Fluorescence has long been used in biomarker analysis, immunoassays, and diagnostic imaging, including cancer diagnosis [1]. Many small organic fluorescent molecules (<1 kD in molecular weight) that are currently commercially available suffer from several limitations concerning wavelength range, brightness (i.e., extinction coefficient for absorbance × quantum yield of fluorescence), photobleaching (i.e., photostability), and fluorescence self-quenching. Also, the toxicity exhibited by some promising organic fluorophore molecules has limited their application when intended for in vitro and in vivo optical imaging.

Fluorescent micro- and nanoparticle systems have been proposed as a solution to the above-mentioned limitations of small organic fluorescent dyes [2]. When compared to the low absorption coefficients of individual organic fluorophore molecules, fluorescent micro- and nanoparticles often contain multiple fluorophore entities, leading to increased photoluminescent emission. Additionally, their encapsulation into the particle provides with improved stability and reduced photobleaching and toxicity, as illustrated by Raymo and co-workers in recent works [3,4]. Improved biocompatibility can also be obtained by proper selection of the nature of the particle. Moreover, a unique feature of micro- and nanoparticles is the possibility to combine imaging and drug delivery characteristics in the same system to produce theranostic agents for nanomedicine applications [5]. Several fluorescent micro- and nanoparticle systems based on block copolymer micelles and cross-linked polymer networks, quantum dots (QDs), π-conjugated polymers, and dendrimers have been evaluated as optical imaging systems. The polymerization of monomers containing fluorescent moieties has been employed to prepare amphiphilic block copolymers (BCPs) which self-assemble in water to produce fluorescent micelles [6]. Also, decoration of non-fluorescent BCPs with fluorescent dyes has been carried out to produce red, green, and blue photoluminescent (PL) emission via crystallization-driven self-assembly, allowing even white light PL emission [7]. Fluorescent cross-linked polymer networks have been typically prepared through direct free radical cross-linking oil-in-water emulsion polymerization [8]. QDs which are inorganic nanocrystals with excellent fluorescence properties (extinction coefficients ×10 to ×100 of those of organic fluorophobe dyes) have been fabricated by a variety of methods [9]. Different fluorescent π-conjugated polymers [10] and dendrimers [11] have also been synthesized and evaluated as optical imaging systems. 

**Figure 1:**
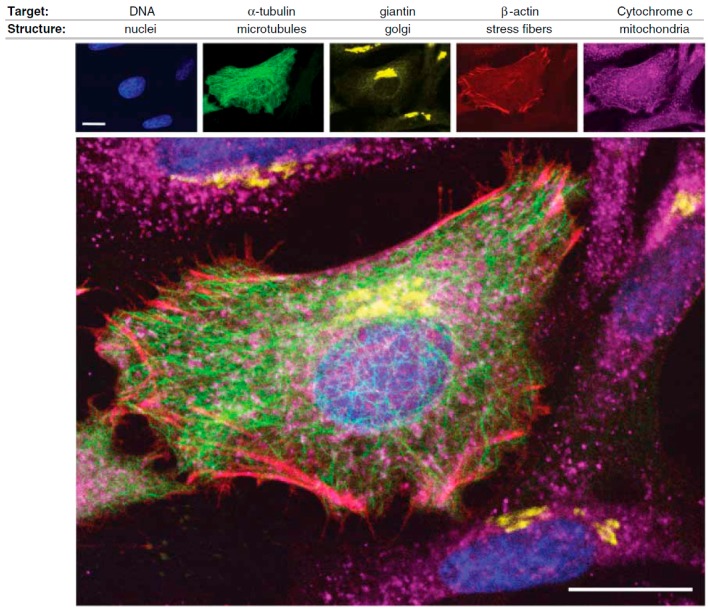
Illustration of the application of complementary fluorophores for the selective detection of different targets in *HeLa* cancer cells: DNA of nuclei (blue color), α-tubulin of microtubules (green color), giantin of Golgi bodies (yellow color), β-actin of stress fibers (red color), and Cytochrome c of mitochondria (purple color). Scale bars: 20 μm (reprinted from [12] with permission, Copyright American Association for the Advancement of Science, 2006).

A nice example of the current fluorescent toolbox available for assessing protein location and function in cell biology is depicted in Figure 1, in which a variety of targeting methods and fluorophores (organic dyes, QDs, green fluorescent protein) were combined to visualize different cell structures of *HeLa* cancer cells [12].

In spite of the successful use of fluorescent micro- and nanoparticles in some optical imaging applications, certain problems remain to be solved, since the large size of these particles (often >10 nm) prevents efficient traversal of intact membranes in cells. Additionally, an appropriate tuning of the ultraviolet-visible (UV-vis) absorption and photoluminescence emission wavelengths is sometimes difficult or even impossible. Moreover, in vivo accumulation of large particles in the body is a real problem for some of these systems.

In recent years, a new type of polymer nanoparticles with a very small size (as small as 3 nm) has been developed [13,14,15,16,17,18,19,20,21,22,23,24,25,26,27,28,29,30,31,32], and several methods to endow these single-chain soft nano-objects so-called single-chain polymer nanoparticles (SCNPs) with fluorescent characteristics have been reported [13]. SCNPs are prepared through the folding/collapse of individual polymer chains by means of intramolecular cross-linking driven by covalent bonds or reversible interactions [21,22,23,24,25,26,27,28,29]. The molecular weight of the SCNP precursor polymer and its functionalization degree are essential parameters that control SCNP size, in addition to the nature of the interactions employed to perform the folding/collapse and solvent quality (good solvent, selective solvent) [14,15]. In this sense, the folding of a linear synthetic polymer to a collapsed state provides with one (or more) denser local packaging zone(s) where fluorophore molecules can be efficiently accommodated (see below for details) [16]. Concerning the morphology of SCNPs in solution, two limiting conformations can be obtained by current synthetic methods: a sparse morphology resembling that typical of intrinsically disordered proteins (IDPs) and a globular morphology as often found in enzymes [18]. 

**Figure 2:**
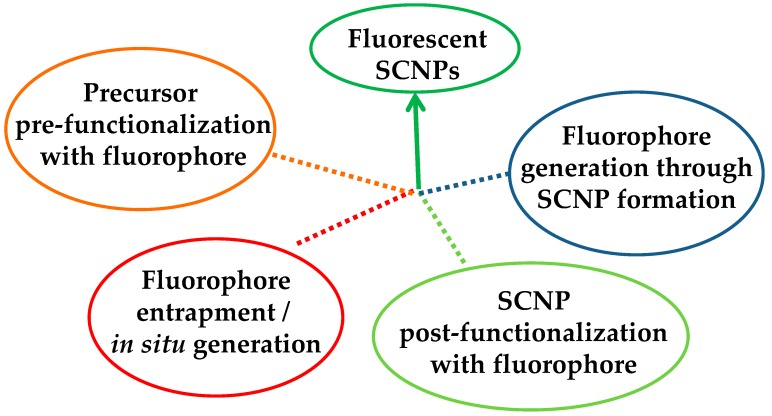
Different strategies developed to endow single-chain nanoparticles (SCNPs) with fluorescent properties.

Four different ways have been opened to endow SCNPs with fluorescence properties (Figure 2): (i) “precursor pre-functionalization with fluorophore,” i.e., functionalization of the SCNP precursor polymer with fluorophore molecules before intramolecular cross-linking, (ii) “fluorophore entrapment/in situ generation,” i.e., entrapment of external fluorophore molecules into non-fluorescent SCNPs by taking advantage of the denser local packaging zone(s) of the SCNPs or in situ generation of the fluorophore molecule inside the SCNP, (iii) “SCNP post-functionalization with fluorophore,” i.e., post-functionalization of the SCNPs via chemical reaction with appropriate, complementary reactive fluorophore molecules, and (iv) “fluorophore generation through SCNP formation,” i.e., generation of fluorophore functional groups through intramolecular cross-linking. 

The present review summarizes the recent advances performed in last years for the construction of fluorescent SCNPs through the above methods, showing illustrative examples.

## 2. Fluorescent Single-Chain Nanoparticles: Synthesis Routes

When compared to the development of other fluorophore micro- and nano-particle systems such as block copolymer micelles and cross-linked polymer networks [6,7,8], quantum dots [9], π-conjugated polymers [10], and dendrimers [11], the preparation of fluorescent SCNPs is still in its early infancy [13]. Nevertheless, four different routes have been established to endow SCNPs with fluorescent properties, paving the way to the potential construction of new fluorescent probes with ultra-small size (e.g., 3 nm in diameter), higher brightness, and better photostability than previous particle-based systems.

### 2.1. Precursor Pre-Functionalization with Fluorophore

**Figure 3:**
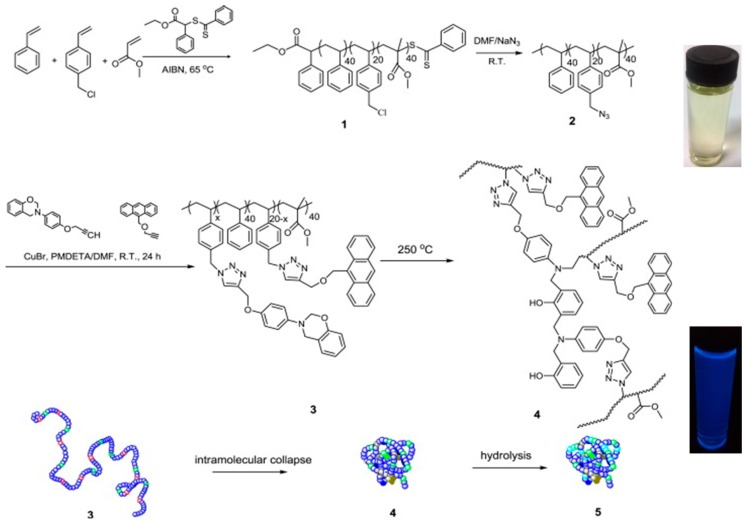
Synthesis of fluorescent SCNPs (**4**) via functionalization of the SCNP precursor terpolymer **2** with reactive (benzoxazine) and fluorescent (anthracene) groups (**3**) followed by intramolecular ring-opening polymerization of the benzoxazine moieties. Hydrolysis of **4** leads directly to water-soluble, fluorescent SCNPs (**5**) with a maximum emission peak centered at 412 nm when irradiated at λ = 367 nm (reprinted from [33] with permission, Copyright Elsevier, 2014).

The synthesis of fluorescent SCNPs through functionalization of the linear precursor polymer with a fluorophore moiety is shown with an illustrative example in Figure 3. In this work by Wang et al. [33], a linear polystyrene (PS)-based terpolymer decorated with azide (–N_3_) functional groups (**2**) was first prepared from a PS-based terpolymer containing chloromethyl functional groups (**1**). Nucleophilic substitution of –Cl groups by –N_3_ moieties was carried out in dimethylformamide (DMF) at r.t. Subsequently, **2** was decorated with reactive and fluorescent groups (benzoxazine and anthracene moieties, respectively) via “click chemistry” to give the functionalized precursor polymer **3**. Intramolecular cross-linking of **3** via benzoxazine ring-opening polymerization (ROP) was carried out at high dilution in dibenzyl ether at 250 °C to give SCNP **4**. A further hydrolysis step under acidic conditions (pH = 3–4) was required to render water-soluble the resulting SCNPs (**5**), which were found to show fluorescence with a maximum emission peak centered at 412 nm when irradiated at λ = 367 nm (see Figure 3). The successful formation of SCNPs with an average hydrodynamic radius of 6 nm was confirmed by a combination of characterization techniques, including size exclusion chromatography (SEC), ^1^H NMR and FTIR spectroscopy, dynamic light scattering (DLS), as well as atomic force microscopy (AFM) and transmission electron microscopy (TEM) measurements.

**Figure 4:**
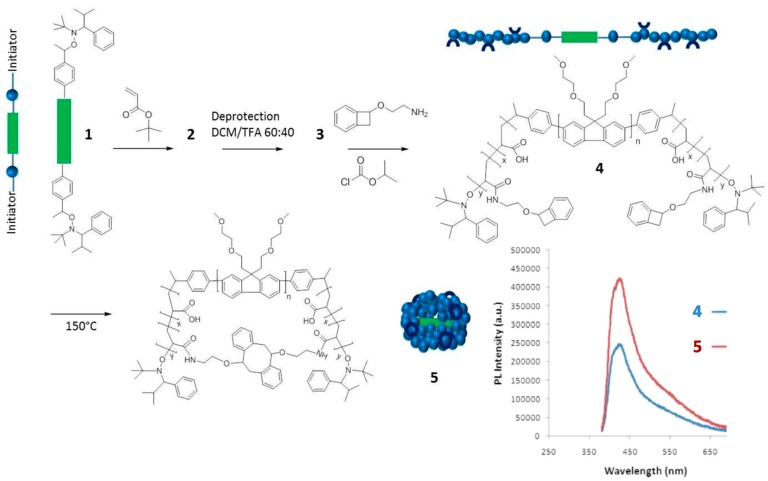
Synthesis of water-soluble fluorescent SCNPs (**5**) from a precursor polymer (**4**) containing a polyfluorene block (depicted in green) via intramolecular single-chain folding/collapse at 150 °C. The values of photoluminescence (PL) quantum efficiency of **4** and **5** (both with maximum PL intensity at 438 nm) were 2.8% and 5.1%, respectively (reprinted from [34] with permission, Copyright American Chemical Society, 2013).

Another example of the “precursor pre-functionalization with fluorophore” method is provided in Figure 4. In this work by Adkins et al. [34], the SCNP precursor polymer **4** was an ABA-type triblock copolymer in which the center block (B) was an ethylene oxide modified polyfluorene segment, and the lateral blocks (A) were polyacrylate segments decorated with low-temperature benzocyclobutene cross-linking groups. Interestingly, the A blocks were synthesized through nitroxide-mediated living radical polymerization by using the fluorescent B block as a telechelic macroinitiator. Characterization of **4** by UV-vis and fluorescence spectroscopy revealed absorption and emission peaks at 370 and 438 nm, respectively, with a quantum efficiency of 2.8%. Upon the folding/collapse of **4** via benzocyclobutene dimerization at high dilution, the resulting SCNPs **5** were found to display UV-vis absorption and fluorescence emission peaks also centered at 370 and 438 nm but with an increased quantum efficiency of 5.1%. This was attributed to the site-isolation effect (i.e., encapsulation) experienced by the fluorescent B block upon intramolecular cross-linking of the A blocks. A similar effect was previously found by Adkins et al. [35] for ABA triblock copolymers containing fluorene and fluorene/thiophene center blocks that were soluble only in organic solvents (dichloromethane and cyclohexane). Remarkably, the length of the A block was found to have a significant effect in promoting this site-isolation effect.

The technique of “precursor pre-functionalization with fluorophore” has been also employed by Bai et al. [36] to prepare organic- and aqueous-soluble fluorescent SCNPs with a diameter as small as 5 nm using a fluorescein-functionalized monomer. The SCNPs were prepared by combining the techniques of ring-opening metathesis polymerization (ROMP) and ring-closing metathesis (RCM). Remarkably, these SCNPs were brighter than fluorescein at a similar concentration, and they showed exceptional stability during photobleaching experiments using 470 nm LED in phosphate buffer at pH = 7.4.

Recently, Blasco et al. [37] reported on the synthesis of fluorescent SCNPs based on polythiophene precursor polymers containing photoresponsive groups. The precursor polymer showed UV-vis absorption and fluorescence emission peaks at 450 and 585 nm, whereas the SCNPs showed absorption and emission peaks at 435 and 580 nm, respectively. The shift in the position of the absorption and emission peaks, as well as the observed reduction in emission intensity (ca. 50%), was attributed to a change in the conformation of the rigid polythiophene chain upon folding/collapse to nanoparticle. 

One clear limitation of this synthesis route to fluorescent SCNPs is the increased possibility of fluorophore-fluorophore interactions upon folding/collapse, leading to fluorescence self-quenching.

### 2.2. Fluorophore Entrapment/In Situ Generation

**Figure 5:**
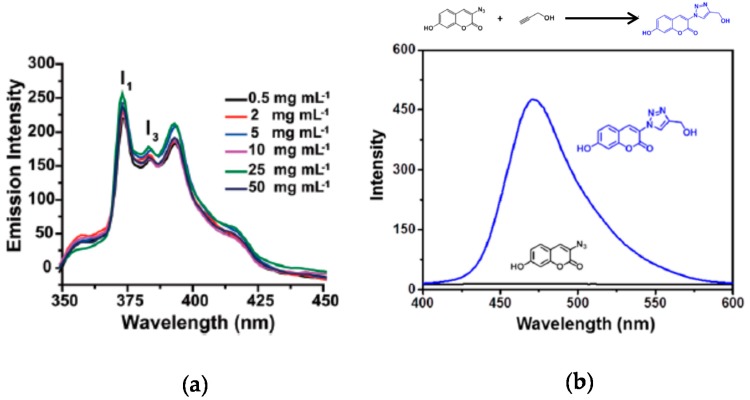
Emission spectra of pyrene sequestered into non-fluorescent SCNPs as a function of the concentration of SCNPs (**a**), and synthesis of a fluorescent compound into non-fluorescent Cu(I)-containing SCNPs via Cu(I)-catalyzed azide-alkyne cycloaddition of 3-azido-7-hydroxycoumarin and propargyl alcohol (**b**) (reprinted with permission from [38], Copyright Royal Society of Chemistry, 2015 and [39], Copyright American Chemical Society, 2015, respectively).

The use of SCNPs as nanocontainers for fluorophore entrapment is another method to endow SCNPs with fluorescent properties. Sometimes, this method has been used to determine the polarity of the dense local packaging zone(s) of the SCNPs upon folding/collapse. As an example, Song et al. [38] applied a pyrene probe to investigate the hydrophobic interior of water-soluble SCNPs prepared through disulfide-based intrachain cross-linking (Figure 5a). It is well-known that pyrene fluorescence depends strongly on the surrounding environment, so the ratio of the intensities between the first and the third peaks (I_1_/I_3_) in the pyrene emission spectrum can range from 1.9 in polar environments to 0.6 in apolar ones (Figure 5a). The disulfide-cross-linked SCNPs showed a ratio I_1_/I_3_ = 1.4, pointing to a moderately hydrophobic interior of the nanoparticle.

Pyrene was also used by Akagi et al. [40] as a fluorescent probe to quantify the hydrophobicity of SCNPs based on a hydrophilic poly(γ-glutamic acid) polymer decorated with hydrophobic L-Phenylalanine (Phe) motifs. SCNPs with high contents of Phe pendants (35 and 42 mol %) were found to display hydrophobic nanodomains in an aqueous solution in which pyrene was solubilized. The same group reported the application of dipyrene to evaluate the rigidity of this kind of SCNPs by fluorescence spectroscopy, recording the fluorescent intensity ratio between the excimer complex (which is present only in low rigidity environments) and the monomer [41]. The rigidity of the inner SCNP structure increased upon the reduction in nanoparticle size and through lyophilization of the samples, which promotes a better close-packing by removing all water molecules from the internal structures.

In situ generation of fluorophore molecules inside SCNPs is another way to endow SCNPs with fluorescent properties, as demonstrated by Liu et al. [39] in a recent work. Amphiphilic SCNPs were prepared by these authors by post-polymerization functionalization of poly(pentafluorophenyl acrylate) as SCNP precursor polymer. Ligand-containing SCNPs capable of coordinating to Cu(I) and Pd(II) were prepared which accelerated azide-alkyne cycloaddition reactions and catalyzed depropargylation reactions, respectively. As a model reaction of fluorophore molecule generation inside SCNPs, 3-azido-7-hydroxycoumarin and propargyl alcohol were chosen as substrates for the Cu(I)-catalyzed azide-alkyne cycloaddition (CuAAC) reaction. The azidocoumarin molecule is non-fluorescent due to emission quenching by the lone pair of electrons from the azido moiety. However, after forming the triazole ring by CuAAC reaction with propargyl alcohol, the lone pair of electrons becomes localized activating the fluorescence (Figure 5b). Fluorescent spectroscopy was used to investigate the kinetics of the CuAAC reaction with different Cu(I)-containing SCNPs under dilute substrate conditions. With the most effective SCNPs, the fluorescence intensity reached a plateau corresponding to full conversion of azidocoumarin to triazole coumarin within less than 10 min. Interestingly, these reactions were found to proceed in phosphate buffer at physiological pH and at low substrate concentrations, which render this kind of SCNPs promising systems to function in complex media such as cellular environments. 

Independently, Bai et al. [42] developed Cu-containing SCNPs as highly efficient in vivo catalyst to endow cells with fluorescence by using 3-azido-7-hydroxycoumarin and 7-ethynylcoumarin as fluorogenic coumarin derivatives that “light up” upon CuAAC reaction with *p*-ethynylanisole and 2-pycolylazide, respectively. These SCNPs showed the ability to enter cells and perform efficient, biocompatible click chemistry in vivo thus acting as intracellular nanoscale molecular synthesizers of fluorophore molecules. In fact, the conversions obtained were typically >90% in 1 h at r.t. at a concentration of SCNPs of only 1 μM. 

**Figure 6:**
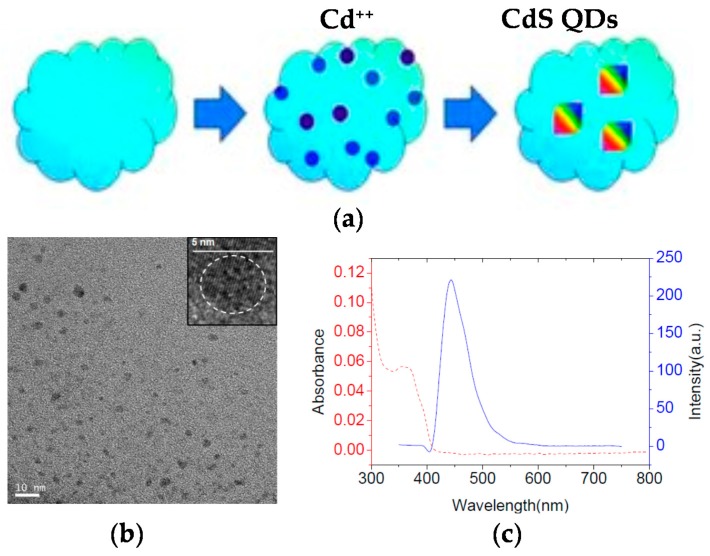
Schematic illustration of the generation of fluorescent CdS quantum dots (QDs) inside non-fluorescent SCNPs (**a**), transmission electron microscopy (TEM) image of the resulting CdS QDs showing an average size of 4.7 nm (**b**), and photoluminiscent emission spectrum (blue) of the CdS QDs displaying bright fluorescence centered at 450 nm (**c**) (reprinted from [43] with permission, Copyright Wiley-VCH, 2012).

The application of non-fluorescent SCNPs as efficient nanoreactors for the synthesis of fluorescent QDs in situ was pioneered by Qian et al. [43] (Figure 6a). Hydrophilic SCNPs synthesized through Bergman cyclization-mediated intramolecular folding/collapse followed by hydrogenolysis on Pd/C were utilized as size-tunable nanoreactors to fabricate and encapsulate QDs in a one-pot reaction. SCNPs were prepared from linear precursors of different size (number-average molecular weight (*M*_n_): 30.3, 62.5 and 115.2 kDa) and functionalization (10 and 25 mol % of reactive enediyne groups). SEC measurements confirmed the successful formation of SCNPs, whereas the 3D morphology of the hydrophilic SCNPs on a mica surface was visualized by AFM. As a proof of concept, photoluminescent zinc sulfide (ZnS) QDs were fabricated and encapsulated in SCNPs of different size. Smaller nanoreactors produce a single QD each (4.1 nm in size) giving to higher emission intensity (quantum yield, QY = 17% at λ = 310 nm), while larger nanoreactors form multiple QDs each, resulting in fluorescence quenching (QY = 2%). To further investigate the generality of the method, cadmium sulfide (CdS) QDs (4.7 nm in size, Figure 6b) were synthesized and encapsulated in the smaller nanoreactors showing bright fluorescence centered at 450 nm (Figure 6c) and excellent values of quantum yield (QY = 45%). Moreover, the use of SCNPs as sacrificial nanoreactors for the synthesis of bright photoluminescent carbon nanodots was further reported by the same group [44].

While a permanent entrapment of QDs inside SCNPs is expected due to the relatively large size of these inorganic nanoparticles, one clear disadvantage of physical entrapment of low molecular weight fluorophore molecules into SCNPs is its reversibility.

### 2.3. SCNP Post-Functionalization with Fluorophore

**Figure 7:**
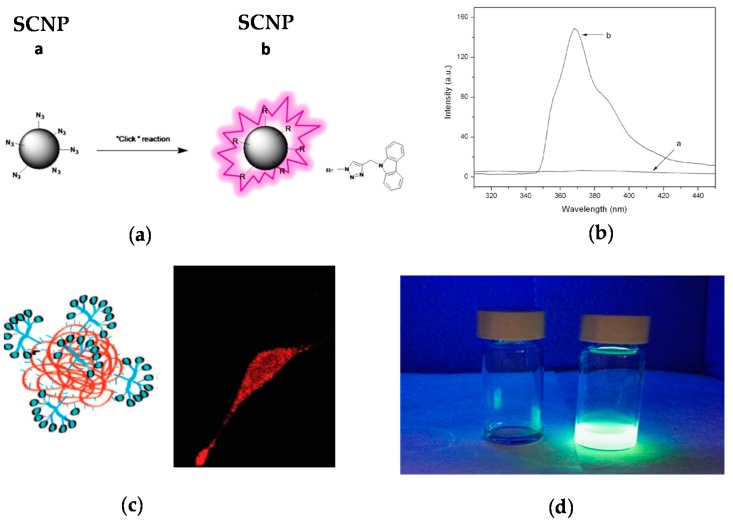
Schematic illustration of the post-functionalization of a SCNP with a fluorophore molecule (**a**), photophysical characterization of the resulting fluorescent SCNP (**b**), and two examples of fluorescent SCNPs decorated with: (**c**) Alexa Fluor 568 dye, and internalized by a fibroblast cell as recorded by confocal fluorescent microscopy, and (**d**) dansylhydrazine (reprinted with permission from [45], Copyright Elsevier, 2014; [46], Copyright American Chemical Society, 2009; and [47], Copyright Royal Society of Chemistry, 2016, respectively).

One strategy to avoid the leaching of entrapped fluorophore molecules from SCNPs is the post-functionalization of the SCNPs via chemical reaction with appropriate, complementary reactive fluorophore molecules, as depicted in Figure 7 [45,46,47]. The first work illustrating a successful post-funtionalization of SCNPs was reported by Jiang and Thayumanavan by using amine-containing SCNPs that were efficiently decorated with pivaloyl groups (>90 mol %) [48]. Post-functionalization of azide-containing SCNPs via the highly-efficient CuAAC reaction was subsequently introduced by Ruiz de Luzuriaga et al. [49]. This approach was followed by Li et al. [45] to react azide-containing SCNPs of 8 nm in size with *N*-propargyl carbazole, rendering fluorescent the initially non-fluorescent SCNPs (Figure 7a). After post-functionalization, the SCNPs showed UV-vis absorption and fluorescence emission peaks at 295 and 365 nm, respectively.

The strategy of SCNP post-functionalization via chemical reaction with reactive fluorophore molecules was exploited by Hamilton and Harth [46] in a seminal work to provide molecular dendritic transporter nanoparticle vectors for efficient intracellular delivery of peptides. In this work, SCNPs were conjugated with both molecular dendritic transporter units to promote intracellular uptake by fibroblast cells, and peptides with cleavable disulfide linkers. Two commercially available fluorescent compounds (Alexa Fluor 568 dye and fluorescein) were used to selectively label the SCNP backbone and the conjugated peptides. Successful uptake and transport of the labeled SCNPs across the cellular membrane was observed by confocal fluorescence microscopy (Figure 7b).

Recently, a variety of reactive fluorophore molecules (dansylhydrazine [47], fluorescein [50], naphthalene [51]) have been successfully conjugated to different (polystyrene [47], polydimethylamino ethyl methacrylate [50], polymethyl methacrylate [51]) nanoparticles by post-functionalization (Figure 7c). This illustrates the versatility of the SCNP post-functionalization technique to endow SCNPs with florescent properties.

### 2.4. Fluorophore Generation through SCNP Formation

**Figure 8:**
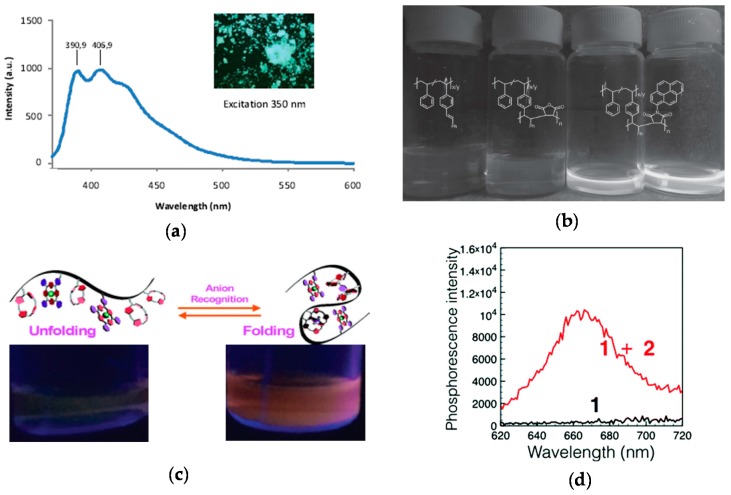
Synthesis of fluorescent SCNPs upon intramolecular cross-linking via formation of fluorophore triazole-benzene-triazole units (inset) and photophysical characterization of the resulting fluorescent SCNP (**a**), fluorescent SCNPs prepared from precursor polymers containing pendant stilbene units through intrachain alternating copolymerization with *N*-(1-pyrene)maleimide (**b**), and phosphorescent SCNPs synthesized by means of anion recognition (**c**); “switch on” of phosphorescence upon anion binding is illustrated in (**d**) (red curve) (reprinted with permission from [52], Copyright Wiley-VCH, 2010; [54], Copyright Wiley-VCH, 2016; and [55], Copyright Royal Society of Chemistry, 2017, respectively).

The technique of fluorophore generation through SCNP formation was first reported by Oria et al. [52], illustrating that fluorescent SCNPs were obtained upon formation of triazole-benzene-triazole units via intramolecular cross-linking. Maximum photoluminescence emission at ca. 400 nm upon excitation at λ = 350 nm was found when azide-containing polystyrene-based SCNPs were reacted with 1,4-diethynylbenzene via CuAAC reaction. The size of these SCNPs was 4.2 nm as determined by DLS measurements. No fluorescent was observed when the triazole-benzene-triazole conjugation was perturbed or absent. A fluorescent microscopy image of these SCNPs in bulk is illustrated in Figure 8a. This work was the first report of fluorescent SCNPs obtained by in situ generation of the fluorophore during intramolecular cross-linking.

Gillisen et al. [53] synthesized bipyridine-containing precursor polymers that fold intramolecularly via π-π interactions into fluorescent SCNPs of ca. 15 nm in size in mixtures of tetrahydrofuran and methylcyclohexane. These SCNPs showed maximum photoluminescence emission at 520 nm upon excitation at λ = 382 nm and were used as fluorescent sensors of metal ions, specifically of Cu(II) due to the strong affinity of the bipyridine units toward Cu(II) leading to strong fluorescence quenching.

Lyon et al. [54] prepared fluorescent SCNPs of 6 nm in size from precursor polymers containing pendant stilbene units via intrachain alternating copolymerization with *N*-(1-pyrene)maleimide (NPM). In the absence of a maleimide monomer, no homopolymerization of the stilbene units to give SCNPs was observed. Hence, the role of the multifunctional NPM monomer was to promote intramolecular cross-linking of the non-fluorescent precursor polymer through alternating copolymerization with stilbene and, concurrently, endowing the resulting SCNPs with fluorescence (Figure 8b).

More recently, phosphorescent SCNPs synthesized by means of simple anion recognition have been reported by Ji et al. [55]. In this work, anion recognition was utilized to control the folding and unfolding of a polymethyl methacrylate-based precursors bearing pendant calix[4]pyrrole and Pt(II)-porphyrin moieties. calix[4]pyrrole was selected as a recognized anion receptor and Pt(II)-porphyrin as a signaling agent due to their bright phosphorescence, long-lived excited-state lifetime and high stability. SCNP formation was carried out in diluted solution using a terephthalate dianion as bifunctional cross-linker, giving to nanoparticles with a diameter as small as 2 nm and a maximum phosphorescence intensity at 670 nm (Figure 8c,d). It is worth of mention that the precursor polymer was not phosphorescent, so the bright phosphorescence of the SCNPs in the latter case was attributed to reduced contact between the Pt(II)-porphyrin moieties and molecular oxygen. Remarkably, the addition of competitive monovalent anions such as F^−^, Cl^−^, H_2_PO_4_^−^, HSO_4_^−^, Br^−^, and NO_3_^−^ gave to a decrease in the phosphorescence intensity due to SCNP unfolding. Phosphorescent SCNPs are hence promising functional nanomaterials for anion recognition.

**Figure 9:**
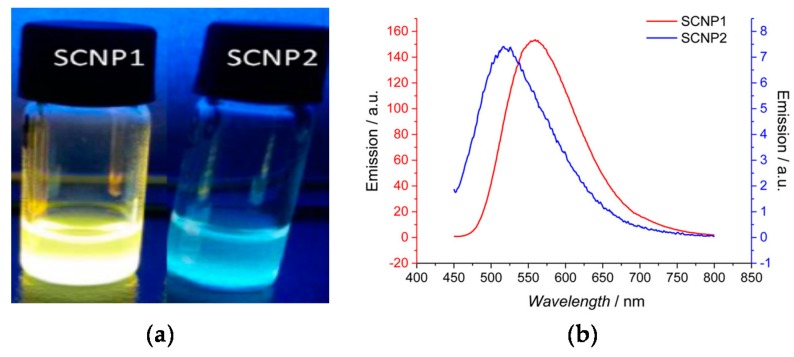
Fluorescent SCNPs produced via nitrile imine-carboxylic acid ligation (SCNP1) as well as nitrile imine-mediated tetrazole-ene cycloaddition (SCNP2) (**a**), and photophysical characterization of SCNP1 and SCNP2 (**b**) (reprinted with permission from [57], Copyright American Chemical Society, 2017).

Different fluorescent SCNPs based on photochemically-activated intramolecular cross-linking procedures have been developed by Barner-Kowollik and coworkers [56,57,58,59] by following the route of fluorophore generation through SCNP formation. In a first work, photoinduced nitrile imine-mediated tetrazole-ene cycloaddition (NITEC) at high dilution in tetrahydrofuran using linear precursor polymers containing protected maleimide (MAL) and tetrazole (TET) pendants was applied to the formation of fluorescent SCNPs. An increased fluorescent intensity was observed upon increasing the MAL content in the precursor polymer. Remarkably, when excited at λ = 415 nm, the maximum of the fluorescence emission band of these SCNPs was placed at 558 nm, which is a wavelength appropriate for biological applications. In a further work by the same group [57], water-soluble fluorescent SCNPs were prepared via two mechanisms based on tetrazole chemistry: NITEC and nitrile imine-carboxylic acid ligation (NICAL) (Figure 9). When compared to fluorescent SCNPs prepared exclusively via NICAL, SCNPs collapsed via NITEC and NICAL showed stronger fluorescence. Recently, new polybutadiene post-functionalization techniques have been introduced by the Barner-Kowollik group [58] to produce fluorescent SCNPs based on intramolecular NITEC chemistry performed in dichloromethane. Moreover, degradable fluorescent SCNPs prepared via intramolecular NITEC chemistry in dichloromethane have been synthetized by this group by utilizing precursor polymers featuring self-immolative azobenzene motifs, which exhibited degradability upon treatment with sodium dithionite [59]. 

## 3. Conclusions

The limitations of conventional fluorophore molecules are mainly related to low brightness, unappropriated wavelength range, rapid photobleaching, and fluorescence self-quenching. Sometimes, toxicity issues limited their use in biological applications. Fluorescent micro- and nanoparticle systems have been proposed as a solution to these limitations since they can contain multiple fluorophore entities leading to brighter photoluminescent emission, improved biocompatibility and stability, and reduced photobleaching and toxicity. Several fluorescent micro-and nanoparticle systems have been developed and evaluated as optical imaging systems (e.g., block copolymer micelles and cross-linked polymer networks, QDs, π-conjugated polymers, and dendrimers). In spite of the successful use of some of these fluorescent micro- and nanoparticles for optical imaging applications, some problems remain to be solved, since the large size of these particles (often >10 nm) prevents efficient traversal of intact membranes in cells, appropriate tuning of UV-vis absorption and photoluminescence emission wavelengths is sometimes difficult (or even impossible), and in vivo accumulation of large particles in the body is a real problem for some of these systems.

In recent years, a new type of polymer nanoparticles, SCNPs of very small size (as small as 3 nm) has been developed, and several methods to endow SCNPs with fluorescent characteristics have been reported. Even if this field is still in its early infancy, fluorescent SCNPs have been prepared through four different methods: (i) “precursor pre-functionalization with fluorophore,” i.e., functionalization of the SCNP precursor polymer with fluorophore molecules before intramolecular cross-linking, (ii) “fluorophore entrapment/in situ generation,” i.e., entrapment of external fluorophore molecules into non-fluorescent SCNPs by taking advantage of the denser local packaging zone(s) of the SCNPs or in situ generation of the fluorophore molecule inside the SCNP, (iii) “SCNP post-functionalization with fluorophore,” i.e., post-functionalization of the SCNPs via chemical reaction with appropriate, complementary reactive fluorophore molecules, and (iv) “fluorophore generation through SCNP formation,” i.e., generation of fluorophore functional groups through intramolecular cross-linking. In this review, the above methods toward fluorescent SCNPs have been discussed in detail, providing many illustrative examples. Future work in this emergent field is expected to be focused on the broadening of the examples of water-soluble fluorescent SCNPs. Simultaneous tuning of the UV absorption and photoluminescent emission characteristics of fluorescent SCNPs is also expected by exploring both traditional and new fluorophores. 

To conclude, a bright future is expected for the application of fluorescent SCNPs to in vitro and in vivo optical imaging as new fluorescent probes with ultra-small size (e.g., 3 nm in diameter), higher brightness, and better photostability than previous systems.
